# Elimination of undifferentiated human embryonic stem cells by cardiac glycosides

**DOI:** 10.1038/s41598-017-05616-2

**Published:** 2017-07-13

**Authors:** Yu-Tsen Lin, Cheng-Kai Wang, Shang-Chih Yang, Shu-Ching Hsu, Hsuan Lin, Fang-Pei Chang, Tzu-Chien Kuo, Chia-Ning Shen, Po-Ming Chiang, Michael Hsiao, Frank Leigh Lu, Jean Lu

**Affiliations:** 1 Graduate Institute of Life Sciences, National Defense Medical Center, Taipei, Taiwan; 2 Genomics Research Center, Academia Sinica, Taipei, Taiwan; 3 Institute of Biochemistry and Molecular Biology, National Yang-Ming University, Taipei, Taiwan; 4 National Institute of Infectious Diseases and Vaccinology, National Health Research Institute, Zhunan, Taiwan; 5 Department of Pediatrics, National Taiwan University Hospital and National Taiwan University Medical College, Taipei, Taiwan; 6 Department of Biotechnology and Laboratory Science in Medicine, National Yang-Ming University, Taipei, Taiwan; 7 Institute of Clinical Medicine, National Cheng Kung University, Tainan, Taiwan; 8 Genomics and System Biology Program, College of Life Science, National Taiwan University, Taipei, Taiwan; 9 National Core Facility Program for Biotechnology, National RNAi Platform, Taipei, Taiwan; 10 Department of Life Science, Tzu Chi University, Hualien, Taiwan

## Abstract

An important safety concern in the use of human pluripotent stem cells (hPSCs) is tumorigenic risk, because these cells can form teratomas after an *in vivo* injection at ectopic sites. Several thousands of undifferentiated hPSCs are sufficient to induce teratomas in a mouse model. Thus, it is critical to remove all residue-undifferentiated hPSCs that have teratoma potential before the clinical application of hPSC-derived cells. In this study, our data demonstrated the cytotoxic effects of cardiac glycosides, such as digoxin, lanatoside C, bufalin, and proscillaridin A, in human embryonic stem cells (hESCs). This phenomenon was not observed in human bone marrow mesenchymal stem cells (hBMMSCs). Most importantly, digoxin and lanatoside C did not affect the stem cells’ differentiation ability. Consistently, the viability of the hESC-derived MSCs, neurons, and endothelium cells was not affected by the digoxin and lanatoside C treatment. Furthermore, the *in vivo* experiments demonstrated that digoxin and lanatoside C prevented teratoma formation. To the best of our knowledge, this study is the first to describe the cytotoxicity and tumor prevention effects of cardiac glycosides in hESCs. Digoxin and lanatoside C are also the first FDA-approved drugs that demonstrated cytotoxicity in undifferentiated hESCs.

## Introduction

Human embryonic stem cells (hESCs) and induced pluripotent stem cells (iPSCs) are human pluripotent stem cells (hPSCs) that have unique self-renewal (ability to replicate almost indefinitely) and pluripotency (ability to differentiate into all cell types of the human body except for placental cells) properties. These abilities make hPSCs promising resources for regeneration therapy^1^. However, substantial challenges remain to be overcome before applying hPSCs to cell therapy. An important safety concern of hPSCs is their tumorigenic risk because these cells can form teratomas after *in vivo* injections at ectopic sites^2, 3^. Thousands of undifferentiated hPSCs residing in millions of differentiated cells are sufficient to induce teratomas in a mouse model^4^. Thus, it is critical to remove all or most of the residue-undifferentiated hPSCs that have teratoma potential before clinical applications using hPSC-derived cells.

There are several strategies to selectively remove hPSCs. These methods include the use of cytotoxic antibodies^5, 6^, specific antibody cell sorting^7–9^, genetic manipulations^10–12^, and pharmacological approaches^13–16^. However, each method has certain disadvantages, such as a high cost (cytotoxic antibodies and specific antibody cell sorting), variation among different lots (cytotoxic antibodies and specific antibody cell sorting)^17, 18^, non-specific binding (cytotoxic antibodies)^18–20^, requirement of genetic manipulation and stable integration of toxic genes (genetic manipulation), and time-consuming procedures (genetic manipulation, specific antibody cell sorting and cytotoxic antibodies). Although many studies have attempted to prevent or block teratoma formation in residual hPSCs, a clinically applicable strategy to eliminate teratoma formation remains to be developed^2, 21^.

In contrast, small molecule approaches have several advantages as follows: these approaches are robust, efficient, fast, simple, and inexpensive, and there is no need to insert genes into cells. Certain small molecules have been shown to inhibit teratoma formation in hPSCs. The inhibitor of stearoyl-CoA desaturase PluriSin #1 prevented teratoma formation^15^. Stearoyl-CoA desaturase is a key enzyme in the biosynthesis of mono-saturated fatty acids and is required for hPSC survival^15^. The N-benzylnonanamide JC011 induced ER stress through the PERK/AT4/DDIT3 pathway^22^. Chemical inhibitors of survivin, such as quercetin and YM155, induced selective cell death and efficiently inhibited teratoma formation^14^. However, neither of these drugs is well defined or approved by the FDA.

In this study, we investigated the roles of cardiac glycosides in human PSCs. Cardiac glycosides (CGs) (also named cardiotonic steroids, CSs) belong to a large family of compounds that can be derived from nature products. Although these compounds have diverse structures, they share a common structural motif. These compounds are specific inhibitors of the transmembrane sodium pump (Na^+^/K^+^-ATPase). CGs inhibit the Na^+^/K^+^-ATPase and then increase the intracellular concentrations of calcium ions^23^. These compounds act as positive inotropic agents, and members of this group have been used in the treatment of heart failure for more than 200 years. One member of this family, digoxin, is still in clinical use^24^. Furthermore, CGs are currently considered to have a potential therapeutic role in cancer therapy^25^. Several studies have reported that CGs play important roles in inducing cell death in several cancer cells^23^. Cancer cells show more susceptibility than cells in normal tissues. The molecular mechanism may be the overexpression of specific alpha subunits of Na^+^/K^+^-ATPase in cancerous cells^26^. These studies indicate that CGs are selective according to the cell type and distinguish between normal cells and transformed cells.

Although cardiac glycosides act as multiple signal transducers, no studies have investigated whether these drugs can eliminate undifferentiated PSCs while sparing their progeny or differentiated cells. In this study, we used digoxin, lanatoside C, bufalin, and proscillaridin A to investigate whether CGs can target hESCs and selectively induce cell death in pluripotent cells. Of these drugs, digoxin and lanatoside C are both FDA approved. Surprisingly, we found that these four drugs efficiently induced cell death in hESCs, but not in differentiated cells or hESC-derived mesenchymal stem cells (MSCs). The *in vivo* experiments also showed that digoxin and lanatoside C successfully prevented teratoma formation.

## Results

### Differential expression of the alpha subunit of Na^+^/K^+^-ATPase in hESCs and hBMMSCs

**Figure 1:**
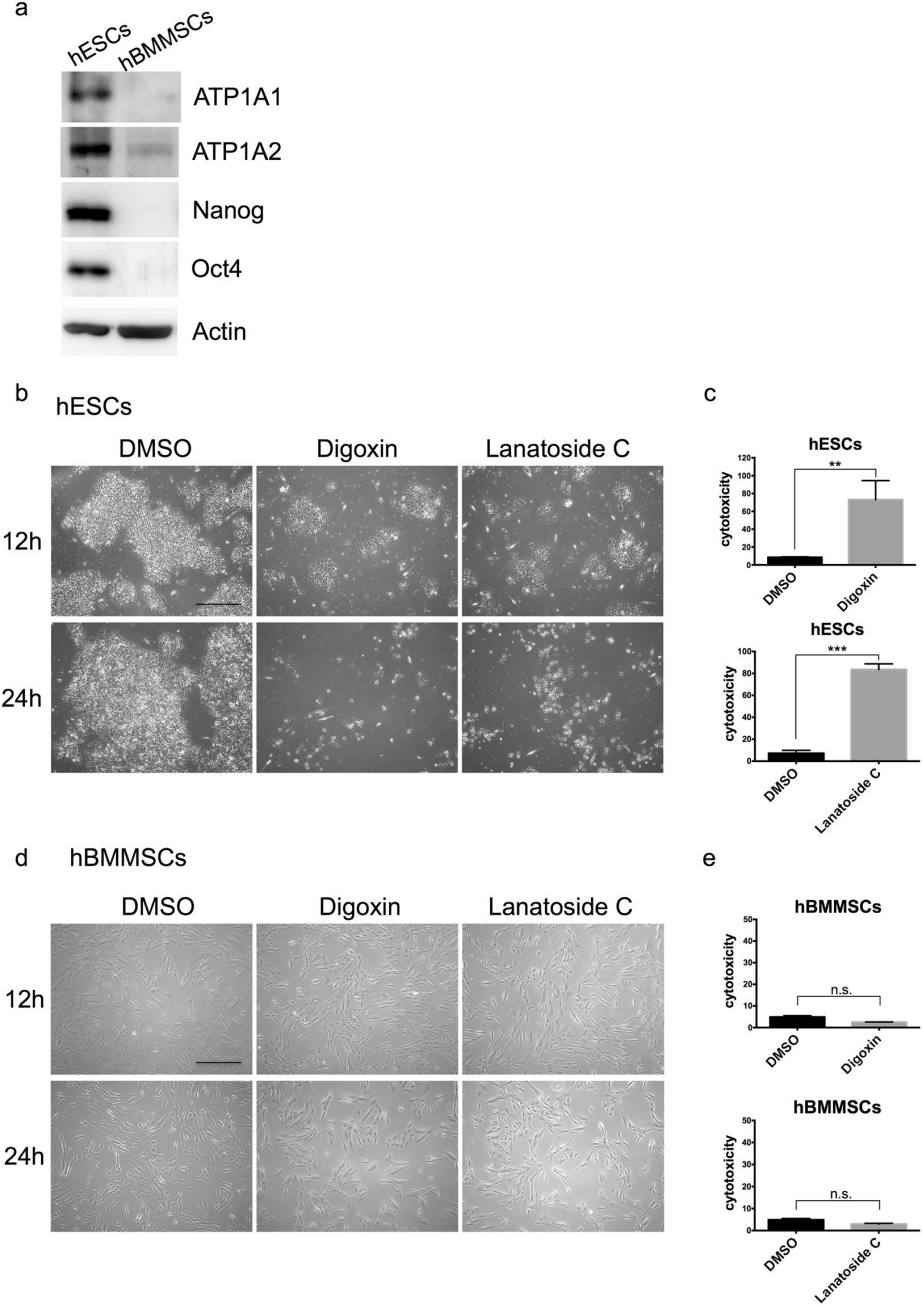
Cardiac glycosides induced cytotoxic effect of in hESCs but not in hMSCs. (**a**) Protein expression levels of the Na^+^/K^+^-ATPase alpha1 and alpha2 subunit in hESCs and hMSCs were detected by western blotting. All unprocessed blot images are presented in Supplementary Fig. S7a. (**b**,**d**) Cell colony and morphology of hESCs and hMSCs under bright field. hESCs and MSCs were treated with DMSO solvent control, 2.5 μM digoxin or 2.5 μM lanatoside C (for 12 hours and 24 hours). Scale bar: 500 μm. (**c**,**e**) LDH release was measured in hESCs and hMSCs to investigate the cytotoxic effect in 96-well culture dishes. DMSO, 2.5 μM digoxin, or 2.5 μM lanatoside C was used to treat the hESCs of hMSCs for 24 hours, and the supernatant was harvested for the LDH detection. The samples were normalized to the DMSO-treated hESCs. ***P < 0.001; **P < 0.01; n.s. not significant. Data are shown as the mean ± SD.

Because not all cancer signals overlap with hESC signals, we determined the expression levels of cardiac glycosides target genes, Na^+^/K^+^-ATPase, to evaluate whether they can eliminate the undifferentiated hESCs. It has previously been reported that cardiac glycosides have anti-cancer effects by targeting Na^+^/K^+^-ATPase^25, 26^. Via a western blot analysis, we found that the hESCs expressed Na^+^/K^+^-ATPase more abundantly than adult stem cells, such as human bone marrow mesenchymal stem cells (hBMMSCs) (Fig. 1a). This finding suggests that hESCs may be more sensitive to cardiac glycosides than hBMMSCs due to their differential expression of Na^+^/K^+^-ATPase.

### Digoxin and Lanatoside C-induced cell death in hESCs but not in hBMMSCs

We investigated whether cardiac glycosides affected the viability of hESCs or other cell types. First, we treated undifferentiated hESCs with digoxin and lanatoside C for 12 hours and 24 hours, respectively. Both digoxin (2.5 μM) and lanatoside C (2.5 μM) induced dramatic cell death in the hESCs (Fig. 1b). To investigate the cytotoxic effect of the cardiac glycosides, we measured the release of LDH in the culture supernatants after the hESCs were treated with digoxin or Lanatoside C for 24 hours (Fig. 1c). Both drugs significantly induced a cytotoxic effect in the hESCs (Fig. 1c). Consistently, in another hESC line, i.e., HUES6, both digoxin (2.5 μM) and lanatoside C (2.5 μM) induced cell death (Fig. S1a) and cytotoxicity (Fig. S1b).

In contrast, digoxin and lanatoside C did not affect the survival of hBMMSCs (Fig. 1d). Both drugs had no cytotoxic effects on the hBMMSCs as measured by the LDH cytotoxic assay (Fig. 1e).

Cardiac glycosides can be divided into two subgroups based on the natural structure of their lactone moiety^23, 26^. Digoxin and lanatoside C belong to a cardenolides subgroup that has butyrolactone^23^. We choose two drugs in the bufadienolides subgroup that have a pyrone ring^23^. We used bufalin or proscillaridin A to treat the hESCs and hBMMSCs. The results were similar to the results of the digoxin- and lanatoside C-treated cells in which bufalin or proscillaridin A induced cytotoxicity in the hESCs but not in the hBMMSCs (Fig. S2).

**Figure 2:**
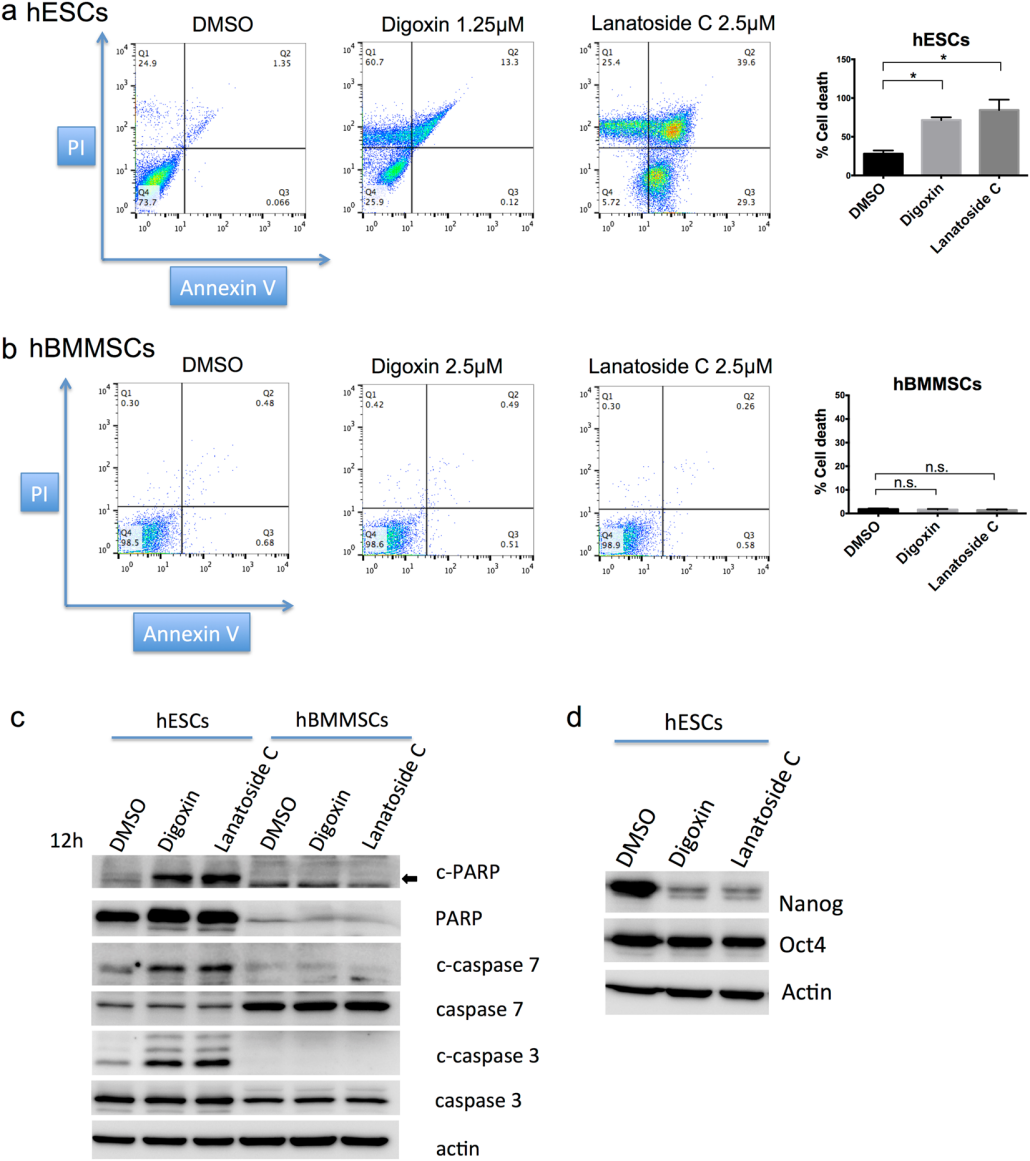
Cell death markers were upregulated in the cardiac glycoside-treated hESCs but not in the hBMMSCs. Cell death was analyzed using hESCs or MSCs. (**a**) hESCs were treated with DMSO, 1.25 μM digoxin, or 2.5 μM lanatoside C for 24 hour. The bar graph represents the statistical results of the FASC data. *P < 0.05; Data are shown as the mean ± SD. Live cells are represented by PI−/Annexin V−; dead cells are represented by PI−/Annexin V+, PI+/Annexin−, and PI+/Annexin+. The bar graph represents the statistical results of the FASC data. n.s. not significant. Data are shown as the mean ± SD. (**b**) hMSCs were treated with DMSO, 2.5 μM digoxin, or 2.5 μM lanatoside C for 24 hours. Cells were stained with PI and Annexin V and subjected to flow cytometry analysis. The bar graph represents the statistical results of the FASC data. n.s. not significant. Data are shown as the mean ± SD. (**c**,**d**) Protein levels of the cleaved form of the apoptotic markers and pluripotent stem cell markers were detected by western blotting. hESCs and hMSCs were treated with DMSO, 2.5 μM digoxin or 2.5 μM lanatoside C for 12 hours. Cells were harvested, and the cleaved and uncleaved forms of PARP, caspase7, and caspase3 were detected. For the detection of the pluripotent stem cell markers, the cells were harvested, and Nanog and Oct4 were detected. All unprocessed blot images are presented in Supplementary Fig. S7b,c.

To determine whether the cytotoxic effect of the cardiac glycosides is selective to hESCs, we also performed a PI/Annexin flow cytometry analysis. After treating the cells for 24 hours, the cell death reached 70% following the digoxin treatment and 82% following the lanatoside C treatment (Fig. 2a). In contrast, more than 98% of the cells were alive in the digoxin- or lanatoside C-treated hBMMSCs (Fig. 2b). In addition, we observed increases in the cleaved form of PARP, caspase-3, and caspase-7 in the digoxin- and lanatoside C-treated hESCs (Fig. 2c). In contrast, no cleaved form of the apoptosis markers was detected in the hBMMSCs treated with digoxin or lanatoside C (Fig. 2c). In addition to the induction of cell death, the tumorigenic potential of the remaining of hESCs was abolished upon cell differentiation. After the hESCs were treated with digoxin or lanatoside C for 12 hours, the protein levels of Nanog were downregulated (Fig. 2d). Nanog is a part of the core transcriptional regulatory networks in ESCs. Loss of Nanog in the hESCs can induce extraembryonic lineage differentiation^27^. Nanog has been reported to play important roles in hESC pluripotency and self-renewal^28^. These results suggested that cardiac glycosides induce cell death in hESCs but not in hBMMSCs.

### Differentiation abilities of hBMMSCs into three lineages are not affected by the digoxin or lanatoside C treatment

**Figure 3:**
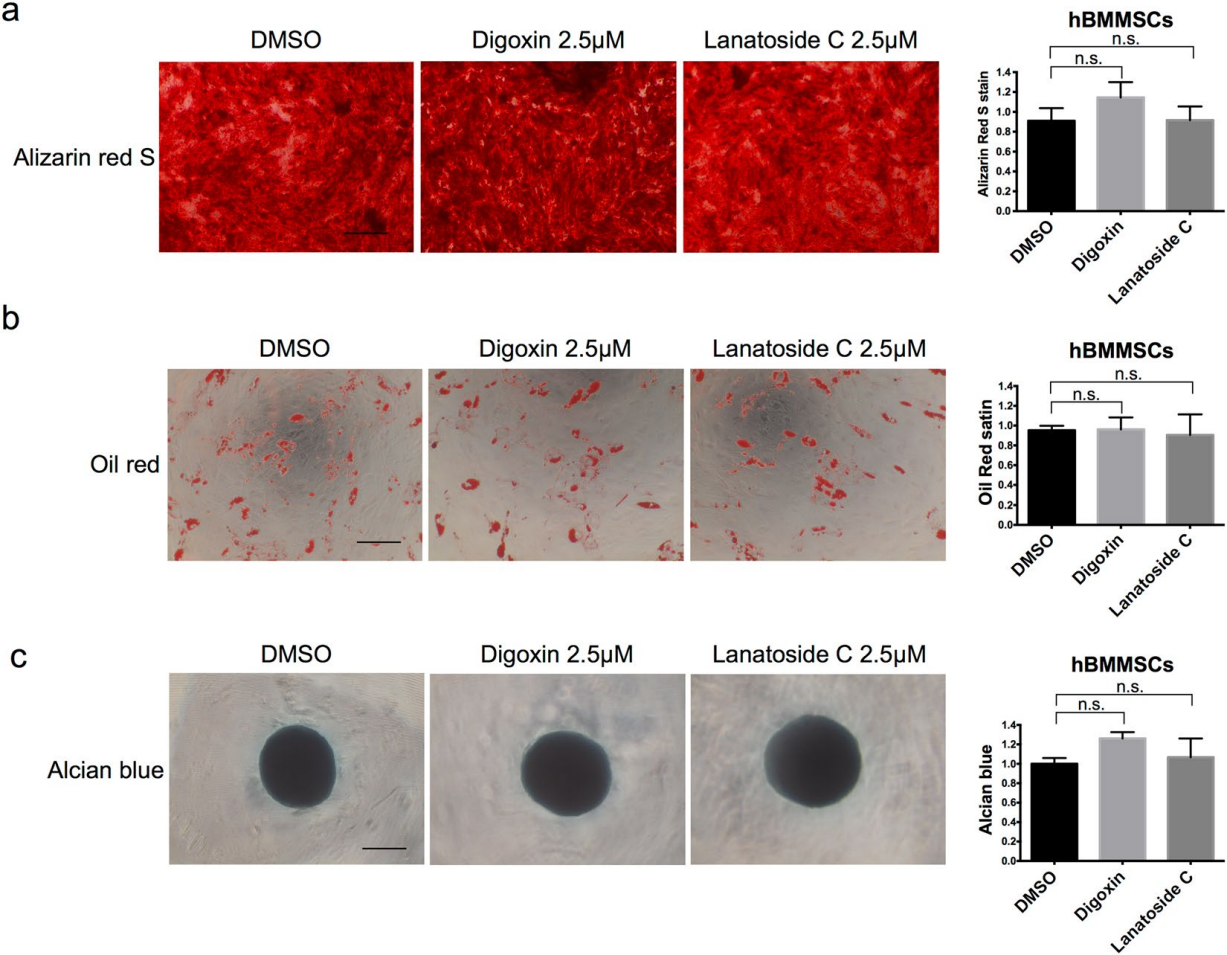
Cardiac glycoside treatment of hBMMSCs did not affect the differentiation abilities. hBMMSCs were treated with DMSO, 2.5 μM digoxin, or 2.5 μM lanatoside C for 24 hours, and the drugs were removed for further differentiation. (**a**) Osteogenic differentiation. Mineralization was stained with Alizarin Red S, and the quantification was performed at O.D 570 nm. (**b**) Adipogenic differentiation. Lipid drop was stained with oil red, and quantification was performed at O.D 510 nm. (**c**) Chondrogenic differentiation. Glycosaminoglycan was stained with Alcian blue, and quantification was performed at O.D 650 nm. Scale bar: 500 μm. n.s. not significant. Data are shown as the mean ± SD.

We demonstrated that the cardiac glycosides did not affect the survival of hBMMSCs. MSCs are multipotent cells that are promising for regenerative medicine. MSCs can be specifically induced into osteoblasts, adipocytes, and cartilage cells^29^. To determine whether the differentiation ability of hBMMSCs is affected by digoxin or lanatoside C, we performed a differentiation assay. Digoxin or lanatoside C was removed after treating the hBMMSCs for 24 hours, and the cells were differentiated into three lineages. Notably, neither digoxin nor lanatoside C affected the differentiation ability of the hBMMSCs into osteoblasts (Fig. 3a), adipocytes (Fig. 3b), and chondrocytes (Fig. 3c). Based on these results, cardiac glycosides do not influence the multipotency of hBMMSCs.

### Digoxin or Lanatoside C did not induce cytotoxic effects in hESC-derived MSCs

**Figure 4:**
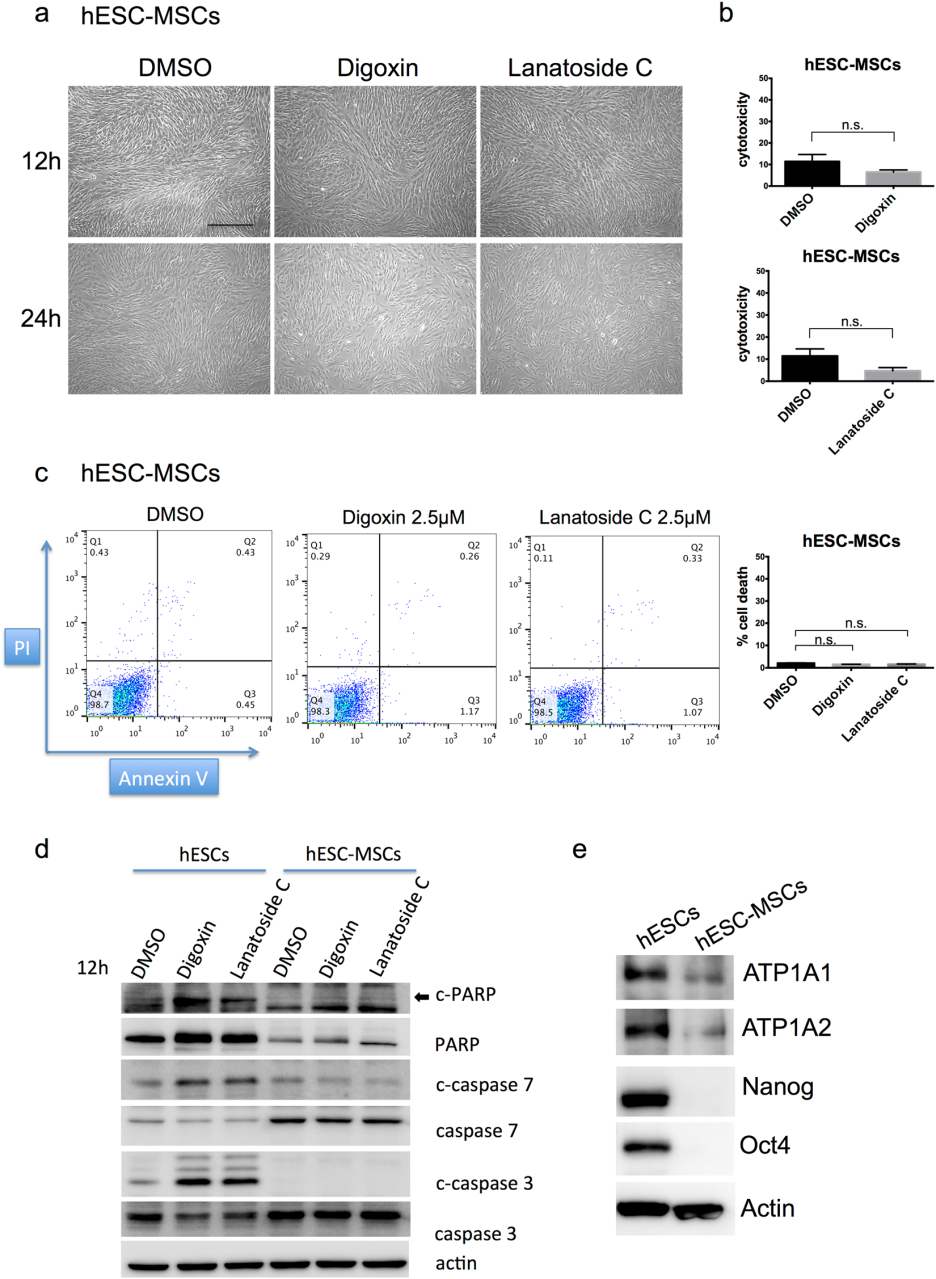
Cardiac glycosides did not affect the cell viability of H9 hESC-derived MSCs. (**a**) Cell morphology of H9 hESC-MSCs under bright field. hESC-MSCs were treated with DMSO solvent control, 2.5 μM digoxin or 2.5 μM lanatoside C for 12 hours and 24 hours respectively. Scale bar: 500 μm. (**b**) LDH release in hESC-MSCs was measured to investigate the cytotoxicity effect in 96-well culture plates. Cells were treated with DMSO or the drugs for 24 hours, and then, the supernatant was harvested for the LDH detection. The samples were normalized against the DMSO-treated hESCs. (**c**) Flow cytometry for the cell death analysis. hESC-MSCs were treated with DMSO, 2.5 μM digoxin or 2.5 μM lanatoside C for 24 hours. Cells were stained with PI and Annexin V, and the cells were analyzed by flow cytometry. The bar graph represents the statistical results of the FASC data. n.s. not significant. Data are shown as the mean ± SD. (**d**) hESCs and hESC-MSCs were treated with DMSO, 2.5 μM digoxin or 2.5 μM lanatoside C for 12 hours. Cells were harvested, and the cleaved and uncleaved forms of PARP, caspase3 and caspase7 were detected by western blotting. (**e**) Protein expression levels of the Na^+^/K^+^-ATPase alpha1 and alpha2 subunits in the hESCs and hESC-MSCs were detected by western blotting. All unprocessed blot images in (**d**) and (**e**) are presented in Supplementary Fig. S8.

To further validate the effects of the cardiac glycosides in hESCs and hESC-derived cell types, we first choose hESC-derived MSCs (hESC-MSCs) as our model. Dr. Xu and colleagues provided a simple and fast method to induce hESCs into hMSCs using a two-step approach^30^ (Fig. S3a). We differentiated the H9 hESCs into MSCs and examined whether the cardiac glycosides affected the viability of the hESC-MSCs. The hESC-MSCs were treated with digoxin and lanatoside C for 12 hours and 24 hours, respectively. Neither digoxin (2.5 μM) nor lanatoside C (2.5 μM) induced cell death in the H9 hESC-MSCs (Fig. 4a). To investigate the cytotoxic effect of the cardiac glycosides, we measured the release of LDH in the culture supernatants after treating the H9 hESC-MSCs with digoxin or Lanatoside C for 24 hours. Neither Digoxin nor lanatoside C affected the survival of the H9 hESC-MSCs (Fig. 4b). In addition to digoxin and lanatoside C, we also found that bufalin or proscillaridin A do not induce cytotoxicity (Fig. S3d). This result is consistent with the effect observed in the hBMMSCs (Fig. S2b).

To investigate whether the cardiac glycosides induced cell death in the H9 hESC-MSCs, PI/Annexin flow cytometry and western blot analyses were performed. After treating the hESC-MSCs with digoxin or lanatoside C for 24 hours, the cell death was less than 2% (Fig. 4c). Consistently, no cleaved form of the apoptosis markers was detected in the hESC-MSCs treated with digoxin or lanatoside C (Fig. 4d). These results suggested that the cardiac glycosides did not induce cell death in the H9 hESC-MSCs. In addition, Na^+^/K^+^-ATPase was abundantly expressed in the undifferentiated hESCs but not in the hESC-MSCs (Fig. 4e), which might demonstrate that the toxicity of the cardiac glycosides is limited to the undifferentiated hESCs.

### Digoxin and lanatoside C did not or slightly induce cell death in hPSCs derived endothelium cells, neurons, or hepatocyte endoderm

We next tested whether digoxin and lanatoside C affected other hPSC-differentiated cell types. The mesoderm lineage of CD34^+^/CD144^+^ hiPSC-derived endothelial cells was obtained from Dr. Chiang and his colleagues (National Cheng Kung University, Tainan, Taiwan) (Fig. S4a). Undifferentiated hiPSCs and hiPSCs-derived endothelial cells were exposed to digoxin or lanatoside C for 24 hours. After the treatment, digoxin and lanatoside C induced cytotoxicity in the hiPSCs (Fig. S4b), but the hiPSC-derived endothelial cells remained alive (Fig. S4c). We differentiated the H9 hESCs into TUJ1-positive neurons that belong to the ectoderm (Fig. S4d) and then treated these hESC-neurons with digoxin or lanatoside C for 24 hours. Digoxin and lanatoside C did not induce cytotoxicity in the neuronal cells (Fig. S4e). In addition, we differentiated the hESCs into AFP-expressing hepatocyte endoderm, which belongs to the endoderm (Fig. S4f). The results showed that the drugs slightly, if at all, induced any cell death in the hepatocyte endoderm cells (less than 10%) (Fig. S4g). Based on the above mentioned results, digoxin and lanatoside C might specifically induce cell death in undifferentiated hPSCs but not in their differentiated progeny.

### Digoxin and lanatoside C prevent teratoma formation in NSG mice

**Figure 5:**
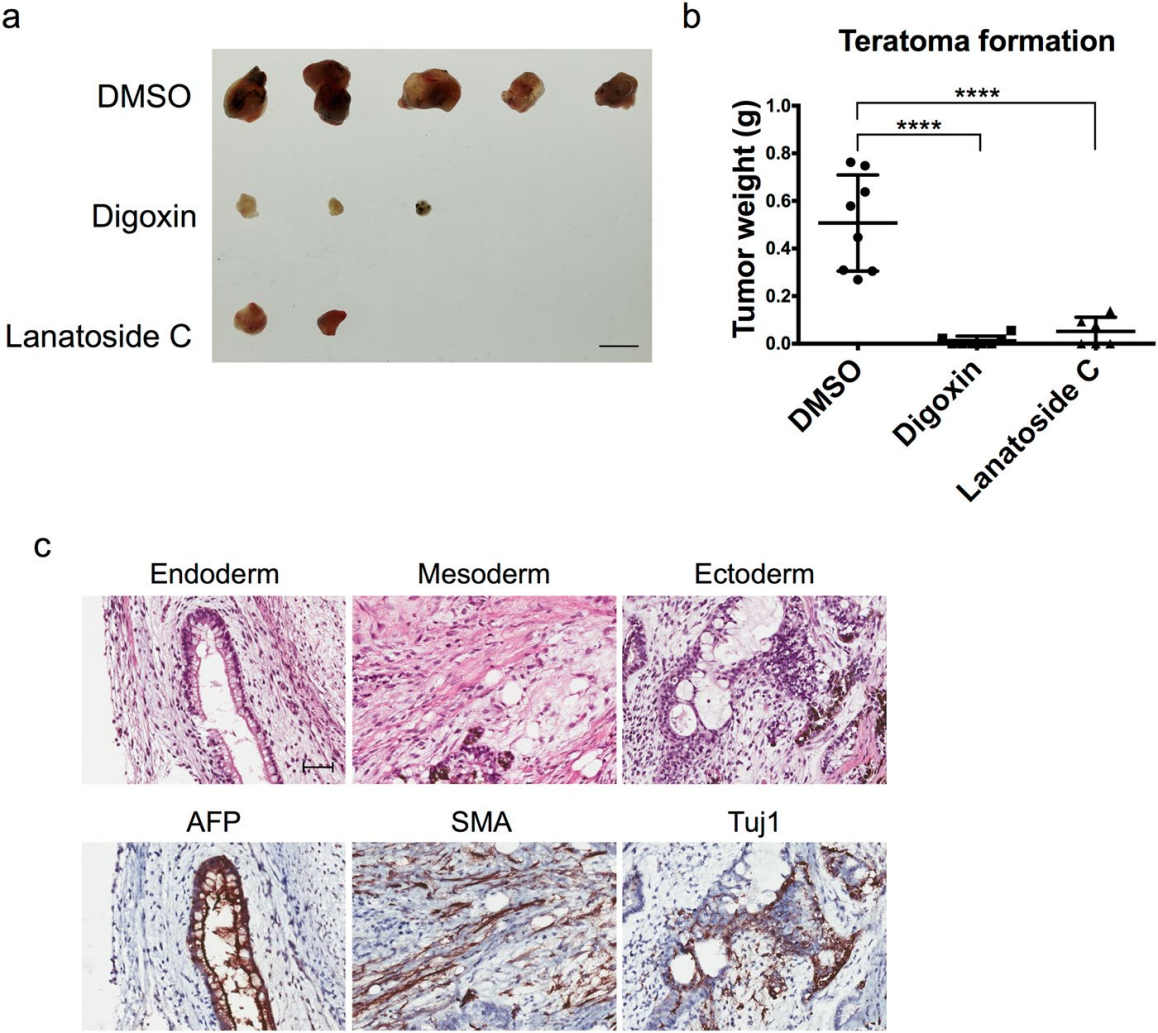
Cardiac glycosides treatment prevented teratoma formation in hESCs in NSG mice. Tumors from NSG mice transplanted with DMSO-, digoxin-, or lanatoside C-treated cells for two months (n = 8 in each group). Scale bar: 10 mm. (**b**) Quantification of the teratoma weight. Dot: DMSO; Square: digoxin; Triangle: lanatoside C. ****P < 0.0001. Data are shown as the mean ± SD. (**c**) These images show a DMSO-treated hESC-derived teratoma. These teratoma paraffin sections were stained with H&E staining (top panel) and IHC staining of three lineage markers (bottom panel). AFP (alpha-fetoprotein): endoderm marker. SMA (smooth muscle actin): mesoderm marker. Tuj1 (beta-III tubulin): ectoderm marker. Scale bar: 50 μm.

To investigate whether hESCs treated with cardiac glycosides lose their ability to form teratomas, hESCs were treated with DMSO, digoxin or lanatoside C and were transplanted into NSG mice individually for xenograft. We found that the tumor weight was significantly decreased in the cardiac glycoside drug-treated group (Fig. 5a and b). Thus, digoxin and lanatoside C severely hampered most of the tumor formation ability in the hESCs. Teratoma formation in the DMSO-, digoxin-, or lanatoside C-treated hESCs was shown to contain all three germ layers (Figs 5c and S5). These results demonstrate the pluripotent ability of these hESCs. We demonstrated that the cardiac glycosides efficiently prevented tumor formation *in vivo*.

Finally, to investigate whether digoxin- or lanatoside C-treated hBMMSCs remain *in vivo*, we constructed GFP overexpressing hBMMSCs. The GFP overexpressing hBMMSCs did not form tumors in the NSG mice under the kidney capsules (Fig. S6a). The GFP-hBMMSCs remained at the graft site, which was observed by GFP IHC staining (Fig. S6a). Then, a mixture of hESCs and hBMMSCs that were treated with the drugs was injected into NSG mice under the kidney capsules. The tumor area was also significantly inhibited in the digoxin- or lanatoside C-treated groups (Fig. S6b), and we still observed GFP-positive hBMMSCs (Fig. S6c). These results demonstrated that digoxin- and lanatoside C-treated hBMMSCs remained in the NSG mice under the kidney capsules.

## Discussion

In cell therapy, residual undifferentiated ESCs or iPSCs in their differentiated progenies raise concerns regarding the safety (teratoma) of using PSC-derived cells. The tumorigenic ability of undifferentiated PSCs is lost upon terminal differentiation. However, residual undifferentiated PSCs must be removed prior to the application of ESC and iPSC cell therapy^31^. In this study, our data demonstrated the cytotoxicity effect of cardiac glycosides in hESCs (Figs 1b,c, 2a,c and S1). This phenomenon was not observed in the hBMMSCs (Figs 1d,e and 2b,c). Most importantly, these drugs did not affect the stem cells differentiation abilities (Fig. 3). A similar effect of cardiac glycosides was shown in the hESC-derived progeny. The viability of the hESC-MSCs, hESC-neurons and hiPSC-endothelial cells were also not affected by digoxin and lanatoside C (Figs 4 and S4). Furthermore, the *in vivo* experiments showed that digoxin and lanatoside C efficiently prevented teratoma formation (Fig. 5). For the first time, our work described the cytotoxic effect and tumor prevention capabilities of cardiac glycosides in hESCs. Digoxin and lanatoside C are also the first FDA approved drugs that have been shown to have cytotoxicity in hESCs.

Cardiac glycosides are rich in many plants, such as the Digitalis species, and they are also extensively found in animal species, mainly in toads^25^. There are only a few reports describing the differentiation roles of cardiac glycosides in PSCs or PSC-derived cells. A high-throughput screening assay of small compound libraries revealed that cardiac glycosides, i.e., cymarin (CYM) and sarmentogenin (SRM), promoted the early differentiation, but not cytotoxicity, of hESCs^32^. Treating hESCs with CYM and SRM inhibited OCT4 expression and induced SOX17 expression^32^. A previous report has shown that digoxin and lanatoside C may reduce TDP-43 protein aggregation in iPSC-derived neurons in sporadic amyotrophic lateral sclerosis (sALS) patients^33^. Change in TDP-43 protein expression and subcellular localization is the most important pathology in sALS^34^. Therefore, these compounds have potential neuroprotective properties. In our present work, we further demonstrated that cardiac glycosides, such as digoxin and lanatoside C, are also involved in the cytotoxic effect and pluripotency.

Cardiac glycosides have been used in the clinic for the treatment of cardiac diseases for a long time. The anti-cancer role of cardiac glycosides is rather novel^23, 35^. Digoxin was found to inhibit several types of cancer cells, such as breast cancer, lymphoma, melanoma, myeloma, and small cell lung cancer^36–38^. Lanatoside C was also demonstrated to inhibit tumor growth, such as colorectal cancer and human hepatocellular carcinoma^39–41^. For synergistic cancer therapy, lanatoside C combined with TRAIL-secreted neural stem cells can target glioblastoma^42^. Thus, cardiac glycosides induce cell death in cancer cells. Cardiac glycosides are well known as a group of Na^+^/K^+^-ATPase specific inhibitors. Changes in the expression levels of Na^+^/K^+^-ATPase subunits were shown in various cancers^23^. The Na^+^/K^+^-ATPase alpha1 subunit was overexpressed in non-small cell lung cancer (NSCLC) cell lines^43^. Inhibiting the expression of the alpha1 subunit impaired proliferation and migration in NSCLC cell lines^43^. A recent study has shown that human NSCLC cells overexpressed the alpha1, alpha2, or alpha3 subunits of Na^+^/K^+^-ATPase and were induced cell death by cardiac glycosides (i.e., digoxin and ouabain)^44^. In our data, the expression levels of the alpha1 and alpha2 subunits of Na^+^/K^+^-ATPase were significantly higher in the hESCs than in the hMSCs or hESC-derived progeny (Figs 1a and 4e). Cardiac glycosides may induce cytotoxicity in hESCs through Na^+^/K^+^-ATPase, which is similar to cancer. More details need to be investigated.

Another possible mechanism of cardiac glycosides is a BCL-2 family anti-apoptotic protein, MCL-1. MCL-1 was demonstrated to be an essential and universal target of cardiac glycosides, and cardiac glycosides cause a downregulation of the MCL-1 protein in various human adherent and non-adherent cancer cells^45^. In another paper, MCL-1 was reported to be more highly expressed in undifferentiated hESCs than in differentiated cells^46^. The authors demonstrated that the loss of Mcl-1 induced cell death in H9 hESCs and suggested that Mcl-1 was critical for hESC survival. Thus, it is also possible that cardiac glycosides induced cell death in the H9 cells by downregulating MCL-1.

To overcome the risk of teratoma formation in regenerative medicine, several strategies have been proposed^47, 48^. Antibody-sorting and cytotoxic antibody strategies may be simple, but their efficiency is limited due to single-cell dissociation requirements or antibody batch variations^7, 17, 18, 49^. The cost of these approaches is also high. Another strategy is based on the genetic manipulation of traceable target cells^10, 12, 50, 51^, but these methods are laborious and expensive. Most importantly, insertion mutagenesis is a biosafety threat in the clinical use of such genetically altered cells^48^. Chemical ablation strategies are rapid, robust and efficient, and they are also the most cost-effective methods. Chemical approaches do not require cell sorting and any genetic manipulation.

In addition to small molecule approaches, a study has used metabolic selection to enrich PSC-derived cardiomyocytes^13^. The authors provided an interesting method to purify cardiomyocytes from PSCs using glucose-depleted and lactate-rich culture conditions. This method is very suitable for generating high purity cardiomyocytes. However, other cell types might need more tests to determine the cell type specific metabolism. This strategy is attractive but can be applied in only a very few cell types. We used CG drugs to eliminate undifferentiated hESCs, and the drugs did not affect the survival of several different cell types (i.e., MSCs, endothelium cells, neurons). Since CGs is easy to purchase and are cost efficient, this method may be convenient for applications in the future. Digoxin and lanatoside C are potent small molecules that inhibit tumorigenic hESCs in culture as shown by the teratoma formation assay (Fig. 5). The expression levels of the Na^+^/K^+^-ATPase subunits were different between the cancer and normal cells or tissues^23^. Furthermore, our data suggested that the expression levels of the Na^+^/K^+^-ATPase subunits were also different between the undifferentiated and differentiated cells (Figs 1a and 4e). In this study, we revealed a novel application of cardiac glycosides that may improve the major concern of hPSCs cell therapy by preventing teratoma formation.

## Materials and Methods

All methods were performed in accordance with the relevant guidelines and regulations. All experiments were approved by Human Subject Research Ethics (AS-IRB02-106069) and Institutional Animal Care & Utilization Committee (IACUC, 14-03-684), Academia Sinica (Taipei, Taiwan). All culture medium and supplements unless otherwise specified, were obtained from ThermoFisher Scientific (Wilmington, DE, USA). All chemicals unless otherwise specified, were brought from Sigma (St. Louis, MO, USA).

### Cell lines and culture conditions

The hESC line H9 was purchased from WiCells (Madison, WI, USA)^52^. Another hESC line, HUES6, was kindly provided by Dr. Douglas A. Melton (Harvard University, Boston, MA, USA)^53^. Cells were maintained on the feeder cells and cultured in Dulbecco’s modified Eagle’s medium (DMEM)/F12 supplemented with 20% knockout serum replacement, 2 mM L-glutamine, 1% nonessential amino acids, 4 ng/mL human bFGF, and 0.1 mM 2-mercaptoethanol. For the feeder cells culture, C57BL/6 mouse embryonic fibroblasts (MEF) were cultured in the DMEM with 10% FBS and treated cells with 0.01 mg/ml mitomycin C 2 hours for inactivation. For the feeder-free culture, hESCs were seeded on the Matrigel Matrix (BD Biosciences, San Jose, CA, USA) coated culture plates and maintained by conditioned medium of MEF (C57BL/6). hBMMSCs were cultured in mesenPRO RS^TM^ kit (Life Technologies, Camarillo, CA, USA). All cells were cultured in a humidified atmosphere containing 5% CO2 at 37 °C.

### Lactate dehydrogenase (LDH) Cytotoxicity assay

The supernatants of cells treated with digoxin 2.5 μM, lanatoside C 2.5 μM, or DMSO solvent control for 24 hours were harvested. The released LDH was measured using CytoTox 96 Non-Cytotoxicity assay according to the manufacture’s instruction (Promega, Southampton, UK). The supernatants and reagents were incubated at room temperature for 20 minutes and then the reaction was stopped by Stop Solution. The absorbance at 490 nm was measured using a plate-reading spectrophotometer (Benchmark Plus Microplate Spectrophotometer System, BIO-RAD, Hercules, CA, USA).

### Western blot analysis

RIPA buffer was used to harvest cell lysates (RIPA buffer: NaCl 150 mM, Tris pH 8.0 50 mM, EDTA pH 8.0 5 mM, NP-40 1.0%, SDS 0.5%, sodium deoxycholate 0.1%). Western blot analyses were performed as previously described with different types of primary antibodies^54^. The primary antibody includes anti-β-actin (A5441; Sigma), anti-Oct4 (sc-9081; Santa Cruz Biotechnology, Santa Cruz, CA, USA), anti-Nanog (3580; Cell Signaling Technology, Danvers, MA, USA), apoptosis antibody kit (9915; Cell Signaling Technology), ATP1A1 (3010; Cell Signaling Technology), and ATP1A2 (16836-1-AP; proteintech^TM^) were used. After reaction at 4 °C overnight, the blots were incubated with goat anti-mouse- or goat anti-rabbit antibody conjugated with horseradish peroxidase (Jackson ImmunoResearch Inc). The chemiluminescent substrate (WBKLS0500; Millipore, Darmstadt, Germany) was used to detect the blots. Fujifilm LAS-4000 (FUJIFILM, Tokyo, Japan) was used to take the images.

### Flow cytometry

For cell death assay, Propidium iodine/Annexin V assay was performed according to the manufacturer’s instruction (Alexa Fluor® 488 Annexin V/Dead Cell Apoptosis Kit; Life Technologies). In brief, live cells were dissociated with trypsin and incubated with Annexin V antibody and PI working solution for 15 minutes. We added 400 μl 1X annexin binding buffer and analyzed the stained cells by FACSCanto^TM^ (Becton Dickinson, Franklin Lakes, NJ, USA). For analyzing the phenotypic signature of MSCs we used a Stemflow^TM^ hMSC analysis kit (BD Biosciences) and FACSCanto^TM^. All flow data was analyzed by FACSDiva^TM^ software (BD Biosciences) and FlowJo^TM^ (FlowJo, LLC, Ashland, USA).

### hESC-derive MSCs

According to the report from Dr. Xu and colleagues^30^, hESCs were cultured with 10 ng/ml BMP4 and 1 μM of A8301 for 5 days. Next, the cells were passaged on the gelatin-coated plate and the culture medium was switched to MSC culture medium [Minimum Essential Medium Eagle Alpha Modification (αMEM) medium supplemented with 20% fetal bovine serum (FBS), l-glutamine (Gluta- MAX), 1x nonessential amino acids]. Cells were expanded within passage 5, and CD73^+^ (11-0793, ThermoFisher Scientific) and CD105^+^ (12–1057, ThermoFisher Scientific) double positive cells were sorted.

### Cell sorting

hESC-derived MSCs were trypsinized and washed with PBS. Then cells were incubated with anti-human CD73 FITC (ThermoFisher Scientific) and anti-human CD105 PE (ThermoFisher Scientific) for 15 minutes at 4 °C. Cells were washed cells with PBS for three times. CD73^+^/CD105^+^ cells were sorted with the cell sorter (BD FACSAria II, BD Biosciences). Sorted cells were maintained in MSC culture medium.

### Osteogenic differentiation and Alizarin Red S staining

BMMSCs were treated with digoxin (2.5 μM), lanatoside C (2.5 μM), or DMSO solvent control for 24 hours. Drugs were removed, and the cells were washed with PBS and cultured in MSCgo™ Osteogenic Differentiation medium (Biological Industries, Kibbutz Beit-Haemek, Israel)^55^. The media changed twice per week for 14–21 days. Next, the cells were fixed with ice-cold 70% ethanol at −20 °C for 1 hour. After washing with water for three times, the cells were then stained with 40 mM Alizarin Red S (ARS) (pH 4.2) at room temperature for 10 minutes. The cells were then washed with PBS for five times. An Olympus CK-40 microscope was used to take the images. For quantification, the dye was extracted with 10% (w/v) cetylpyridinium chloride (Sigma 0732) in sodium phosphate buffer (pH 7.0) for 15 minutes, and the O.D. at 570 nm was measured.

### Adipogenesis and Oil Red O assay

hBMMSCs were treated with digoxin (2.5 μM), lanatoside C (2.5 μM), or DMSO solvent control for 24 hours. Next, drugs were removed, and the cells were cultured in MSCgo™ Adipogenic Differentiation Medium (Biological Industries)^55^. The media was changed every 3–4 days for 8–12 days. After the adipogenic induction, we replaced the MSCgo™ Adipogenic complete medium with MSC maintenance medium for 6–9 days. The cells were carefully fixed with 4% formaldehyde for 1 hour at room temperature, washed with 60% isopropanol, and air-dried. The lipid drops were stained with Oil Red O working solution (30 ml 0.35% oil red solution in isopropanol diluted with 20 ml of distilled water) for 10 minutes. Next, the cells were washed with water 4 times. For quantification, Oil Red O stain was extracted with 100% isopropanol, and the absorbance at 510 nm was detected.

### Chondrogenic induction and Alcian blue assay

BMMSCs were treated with digoxin (2.5 μM), lanatoside C (2.5 μM), or DMSO solvent control for 24 hours. Next, drugs were removed, and 1 × 10^5^ cells were seeded in 96-well U-bottom culture plated. After 24 hours, we changed the complete MSCgo™ Chondrogenic medium (Biological Industries) for 21 days. The media were changed every 3–4 days. Next, the pelleted cells were fixed with 4% formaldehyde for 1 hours, washed twice with ddH_2_O, and stained with a 1% Alcian blue solution in 0.1 N HCl for 30 min. For Alcian Blue elution, we added 8 M Guanidine HCL solution and incubated overnight at RT. The absorbance at 650 nm was detected.

### *In vivo* tumorigenicity assay and immunohistochemistry

hESCs were treated with digoxin (2.5 μM), lanatoside C (2.5 μM), or DMSO control for 24 hours. Approximately 10^6^ treated cells were mixed with 10^5^ MEFs to promote teratoma formation in 50 μl PBS^56^. The cells mixture and 1x Matrigel Matrix was mixed well and the cells were subcutaneously injected into NOD *scid* gamma mice (NSG mice) for 8 weeks. After 8 weeks, animals were sacrificed and teratoma was removed, fixed in 10% formalin, embedded in paraffin and stained with hematoxylin and eosin. H&E stain protocol was modified from previous study^57^. For immunohistochemistry, teratoma sections were blocked using 5% milk for 1 hour, and stained with primary antibody at 4 °C overnight, follow by secondary antibody (Dako, Santa Clara, CA, USA) for 1 hour at RT and DAB enhancer (Dako). Primary antibody: anti-human alpha-1-fetoprotein (A0008, Dako) for endoderm lineage; anti-human smooth muscle actin, clone 1A4 (M0851, Dako) for mesoderm lineage; anti-Tuj1 (MAB1637, EMD Millipore, Darmstadt, Germany) for ectoderm.

### Statistical analysis

All statistical data are presented as the mean ± standard deviation (S.D.) of at least three biological replicates. Statistically significant differences were assessed by t test or One-Way ANOVA, where *p*-value < 0.05 was considered a significant difference.

## Electronic supplementary material

Supplementary Information
