# Supplementary material for: Elimination of undifferentiated human embryonic stem cells by cardiac glycosides
Source: Sci Rep. 2017 Jul 13;7:5289. doi: 10.1038/s41598-017-05616-2 (PMC5509667; doi:10.1038/s41598-017-05616-2)
Supplement: Supplementary file 1 — Supplementary Information [file 41598_2017_5616_MOESM1_ESM.doc]

**Supplementary Information**

**Elimination of undifferentiated human embryonic stem cells by cardiac glycosides**

Yu-Tsen Lin ^1,2^, Cheng-Kai Wang^2,3^, Shang-Chih Yang^2,3^**, Shu-Ching Hsu^4^**, Hsuan Lin^5^, Fang-Pei Chang^2^, Tzu-Chien Kuo^6^, Chia-Ning Shen^2^, Po-Ming Chiang^7^, Michael Hsiao^2^, Frank Leigh Lu^5^, and Jean Lu ^1,2,3, 8,9,10*^

**Supplemental Materials and Methods**

**Supplemental Materials and Methods**

**Differentiation of hiPSC cells into endothelial cells**

hiPSC-derived endothelial cells were prepared according in a previous report^1^. The hiPSCs were maintained in mTeSR 1 medium (STEMCELL Technologies, Vancouver, Canada) on VTN-N coated plates (Thermo, Wilmington, DE, USA). First, for the stepwise induction of differentiation, we performed a mesoderm induction of hiPSCs seeded in mesoderm induction medium for 48 hours (basal medium with 10 mM of Y-27632, 3 mM of CHIR99021, and 2 ng/ml of Activin A; basal medium was composed of 12 g/L of DMEM/F-12, 3.56 g/L of HEPES, 1.742 g/L of sodium bicarbonate, 14 µg/L of sodium selenite, 10.7 mg/L of recombinant transferrin, 19.4 mg/L of recombinant insulin, and 64 mg/L of L-Ascorbic acid 2-phosphate sesquimagnesium salt hydrate). Second, for the mesoderm to endothelium transition, mesoderm cells were reseeded in vasculogenic medium (basal medium with 2 mg/ml of PVA plus 20 ng/ml of hVEGF-A) for an additional 72 hours. All differentiated cells were plated on VTN pre-coated culture plate dishes. The endothelial cells were analyzed on day 5 by flow cytometry staining with the CD31 (PECAM1, ThermoFisher Scientific, Wilmington, DE, USA) and CD144 (CDH5, ThermoFisher Scientific) antibodies.

**Differentiation of hESCs into neurons**

The neuron induction protocol is based on a previous study^2^. Briefly, hESCs were detached by a 1 mg/ml collagenase IV treatment for 1 hour and re-suspended in an embryoid body (EB) medium (a bFGF free hESCs culture medium containing 2 µM dorsomorphin and 2 µM A-83-01b) in non-treated polystyrene plates for 7 days. Then, the EBs were seeded on Matrigel-coated 6-well plates, and the medium was replaced by A neural progenitor cell (NPC) medium consisting of DMEM/F12: Neurobasal = 1:1, 1% N2, 1% B27, 1% NEAA, 1% GlutaMax, 2 µg/ml heparin and 2 µM cyclopamine. The attached EBs were cultured for 14 days with the NPC medium, and the medium was changed every other day. The neural progenitor cells were selected mechanically and then re-suspended on 6 well plate coated with ultra-Low Attachment Surface (Cat. No. 3471, Corning, NY) in the NPC medium. For the neuronal differentiation, suspended neural progenitor spheres were treated with Accutase for 10 min and placed onto poly-D-lysine coated coverslips in the neuronal culture medium, which consisted of Neurobasal medium supplemented with 1% GlutaMax, 1%B27, 10 ng/ml BDNF and 10 ng/ml GDNF. The medium was changed weekly, and the following experiment was performed with the neurons after 3 weeks. The expression of TUJ1 (801202, Biolegend) was assessed in the neural cells via IF staining.

**Differentiation of hESCs into hepatocytes**

This protocol followed a method previously published in *Nature Protocols*^3^. The hESCs were detached by 0.5 mM EDTA (Life Technologies, Camarillo, C, USA), seeded on VTN-N coated culture plates (Corning) and maintained for 48 hours in mTeSR 1 medium. Then, the medium was refreshed daily for all following steps. On days 1-2, the medium was replaced with fresh CDM-PVA (consisting of 0.5 g of PVA (Sigma) dissolved in 250 ml of IMDM/F-12, GlutaMAX (Invitrogen), 250 ml of IMDM (Invitrogen), 5 ml of chemically defined lipid concentrate (Invitrogen), 20 μl of thioglycerol 97% (Sigma), 350 μl of insulin (10 mg/ml; Roche), 250 μl of transferrin (30 mg/ml; Roche) plus Activin A (100 ng/ml; R&D), bFGF (80 ng/ml; R&D), BMP4 (10 ng/ml; R&D), and 10 mM of LY-294002 (Promega). On day 3, the cells were differentiated in RPMI Medium supplemented with Activin A (100 ng/ml) and bFGF (80 ng/ml). On days 4-6, the cells were expanded in RPMI medium supplemented with Activin A (50 ng/ml). On day 7, the cells were passaged and re-plated 105,000 cells/cm2 cells in VTN-N coated culture plates in RPMI containing Activin A (50 ng/ml) and Y-27632 2HCl (10μM Selleck-chem, Houston, TX, USA and Munich, Germany). Then, the cells were maintained in RPMI +Activin A (50 ng/ml) on days 8-11. The cells were differentiated into the hepatocyte endoderm and analyzed following the drug treatments. The expression of alpha-fetoprotein (A0008, Dako, Santa Clara, CA, USA) was analyzed in the hepatocyte endoderm via immunofluorescence staining.

**Immunofluorescence assay**

The immunofluorescence assays were performed as previously described^4^. In brief, the cells were washed with PBS, fixed with 4% formaldehyde solution in PBS for 10 mins and permeabilized by 0.3% Triton X-100 for 10 mins. Then, the cells were stained with primary antibodies overnight at 4°C. After washing with PBS, the cells were incubated with Alexa Fluor® 488 anti-rabbit IgG or anti-mouse IgG for 1 hour at room temperature. The nuclei were stained with 4,6-diamidino-2-phenylindole (DAPI, l μg/ml)

**Lentivirus production and cell infection**

The 293T cells were seeded with 10^6^ cells in 6-well plates for the generation of the lentivirus. After 24 hours, the 293T cells were transfected with 1 μg of pLKO_AS3w.eGFP.bsd, 0.9 μg of pCMVR8.91, and 0.1 μg of pMD.G (National RNAi Core Facility, Taipei, Taiwan) via the TurboFect transfection reagent (Thermo Fisher Scientific). At 24 hours after the transfection, the medium was refreshed with the virus harvest medium, which contained HG-DMEM, 10% FBS and 1% BSA. The hBMMSCs were seeded in 6-well plates and later incubated with the lentivirus (multiplicity of infection = 10) for one day. The cells were selected with 10 μg/ml blasticidin (Thermo Fisher Scientific) the following day. The GFP overexpressing hBMMSCs were then isolated using a cell sorter (FACS AriaTM II, BD Biosciences) and cultured in a medium containing 10 μg/ml blasticidin for the following experiments.

**Kidney capsule transplantation**

Mixtures of approximately 8x10^5^ hESCs and 8x10^5^ hBMMSCs were injected under the kidney capsules into 6-week-old male NSG mice. Five weeks after the transplantation, the animals were sacrificed, and the tissues were removed, fixed in 10% formalin, embedded in paraffin, stained with H&E or were subjected to IHC staining. The staining protocols were described in the materials and method section. Chicken anti-GFP (ab 13970, Abcam, Cambridge, MA, USA) antibody was used for the GFP overexpressing hBMMSCs IHC staining.

1 Wu, Y. T. *et al.* Defining minimum essential factors to derive highly pure human endothelial cells from iPS/ES cells in an animal substance-free system. *Sci Rep* **5**, 1-9, doi:10.1038/srep09718 (2015).

2 Wen, Z. *et al.* Synaptic dysregulation in a human iPS cell model of mental disorders. *Nature* **515**, 414-418, doi:10.1038/nature13716 (2014).

3 Hannan, N. R., Segeritz, C. P., Touboul, T. & Vallier, L. Production of hepatocyte-like cells from human pluripotent stem cells. *Nat Protoc* **8**, 430-437 (2013).

4 Wang, C. H. *et al.* A shRNA functional screen reveals Nme6 and Nme7 are crucial for embryonic stem cell renewal. *Stem cells (Dayton, Ohio)* **30**, 2199-2211, doi:10.1002/stem.1203 (2012).

**
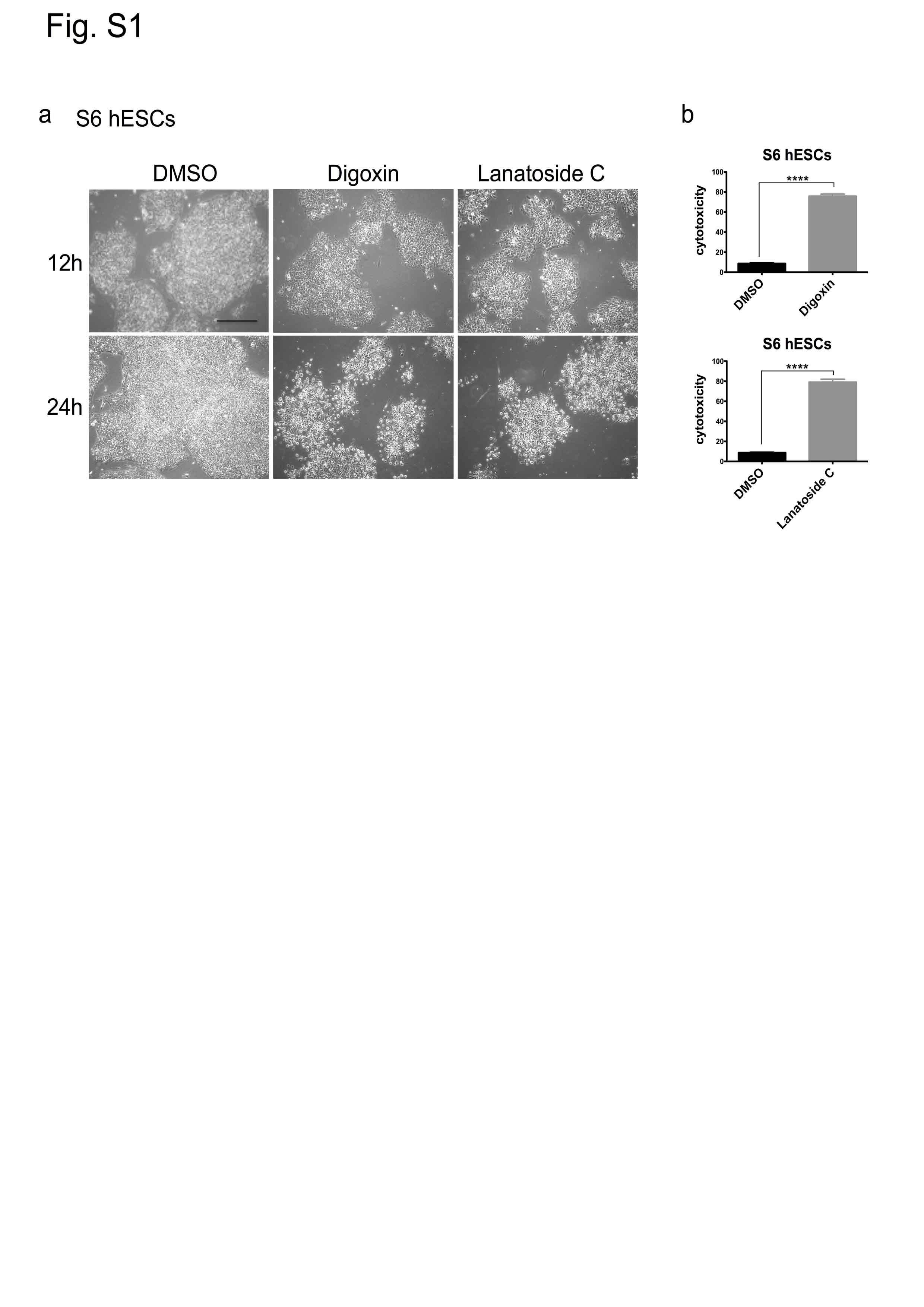
**

**Supplementary Figure 1.** Cardiac glycosides caused cytotoxicity in HUES6 hESCs. (a) Cell colony and morphology of HUES6 under bright field. S6 hESCs were treated with DMSO, 2.5 μM digoxin, or 2.5 μM lanatoside C for 12 hours and 24 hours. Cells became round and died. (b) S6 hESCs were treated with DMSO, 2.5 μM digoxin, or 2.5 μM lanatoside C for 24 hours. The culture supernatant was harvested for the LDH release detection. ***P<0.001. Data are shown as the mean ± SD.

**
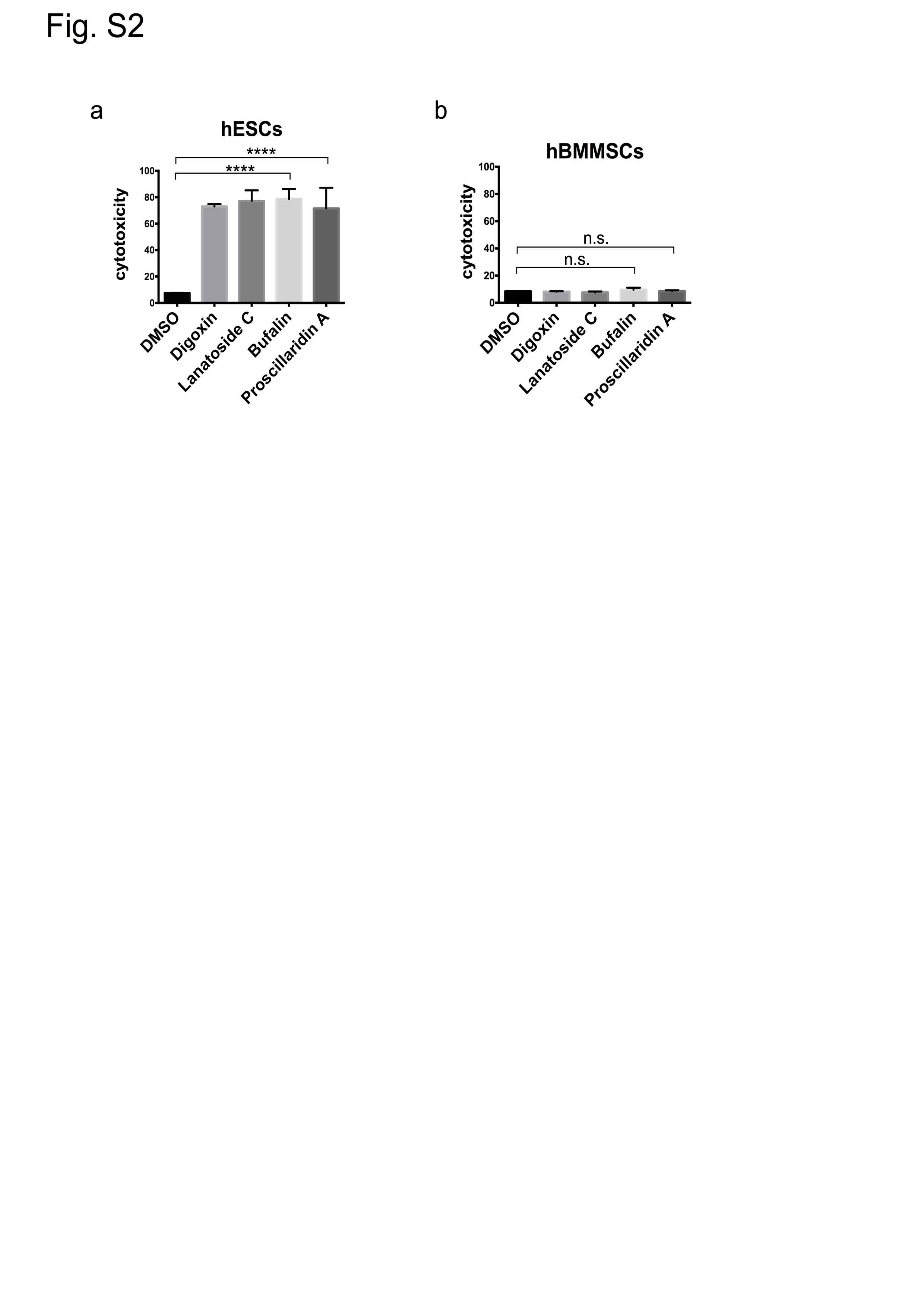
**

**Supplementary Figure 2.** The bufadienolide subgroup of cardiac glycosides, i.e., bufalin and proscillaridin A, induced a cytotoxic effect in hESCs but did not affect hBMMSCs.

(a) hESCs and (b) hBMMSCs were individually treated with different cardiac glycosides at a final concentration of 2.5 μM for 24 hours. The cytotoxic effect was measured by an LDH release assay, and all data were compared with the DMSO solvent control. Cardenolide: digoxin and lanatoside C; bufadienolides: bufalin and proscillaridin A. ****P<0.0001; n.s. not significant. Data are shown as the mean ± SD.

**
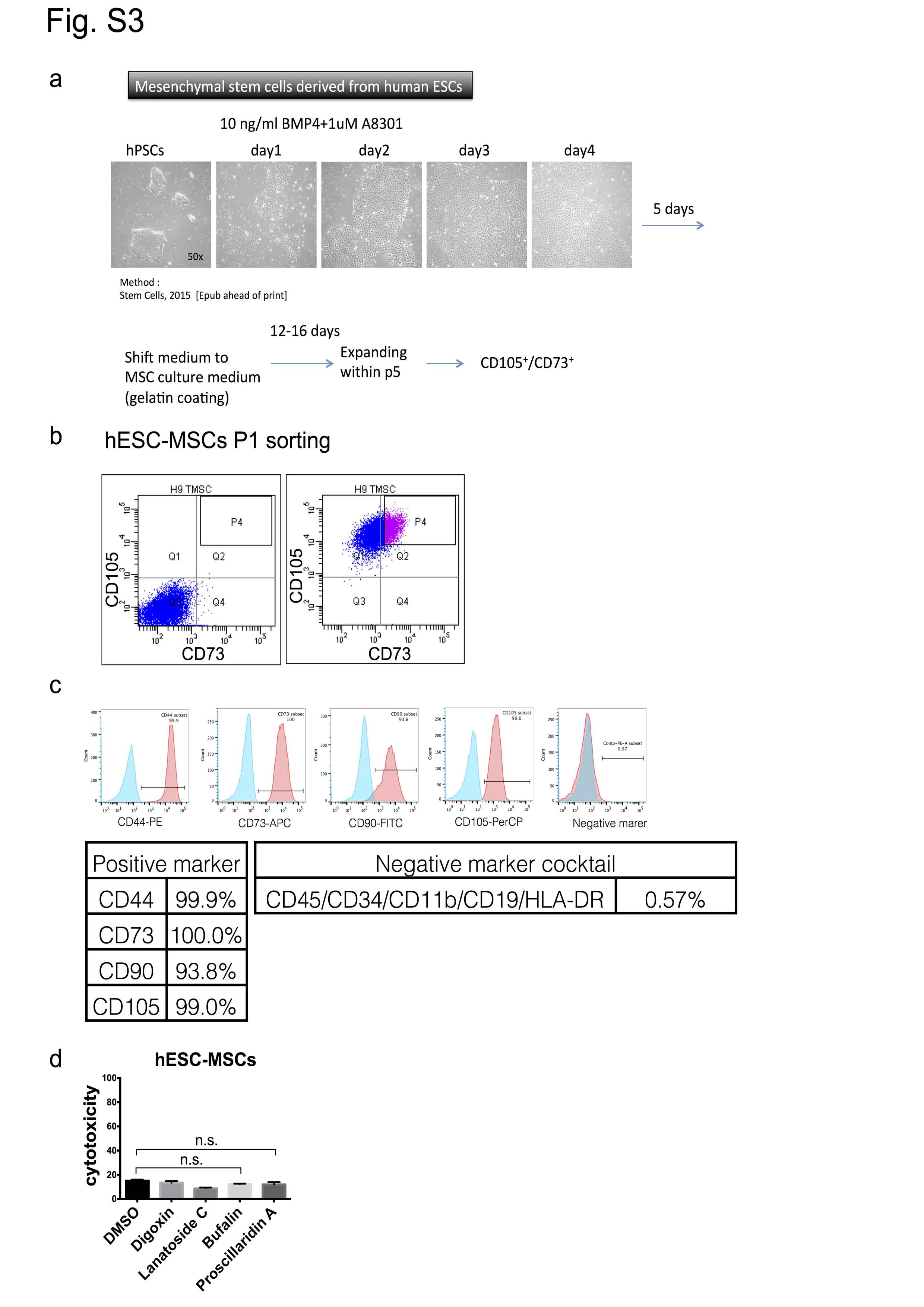
**

**Supplementary Figure 3.** **MSCs are derived from H9 hESCs.** (a) Schematic diagram of the differentiation process**.** (b) The phenotype of passage 1 hESC-MSCs was analyzed and sorted by CD73 and CD105. (c) General MSC-positive and MSC-negative markers were analyzed in the hESC-MSCs. Positive markers: CD73, CD90, CD44, and CD105; negative markers: CD45, CD34, CD11b, CD19, and HLA-DR. (d) hESC-MSCs were individually treated with different cardiac glycosides at a final concentration of 2.5 μM for 24 hours and followed by measuring the amount of released LDH in the cytotoxic assay. n.s. not significant. Data are shown as the mean ± SD.

**
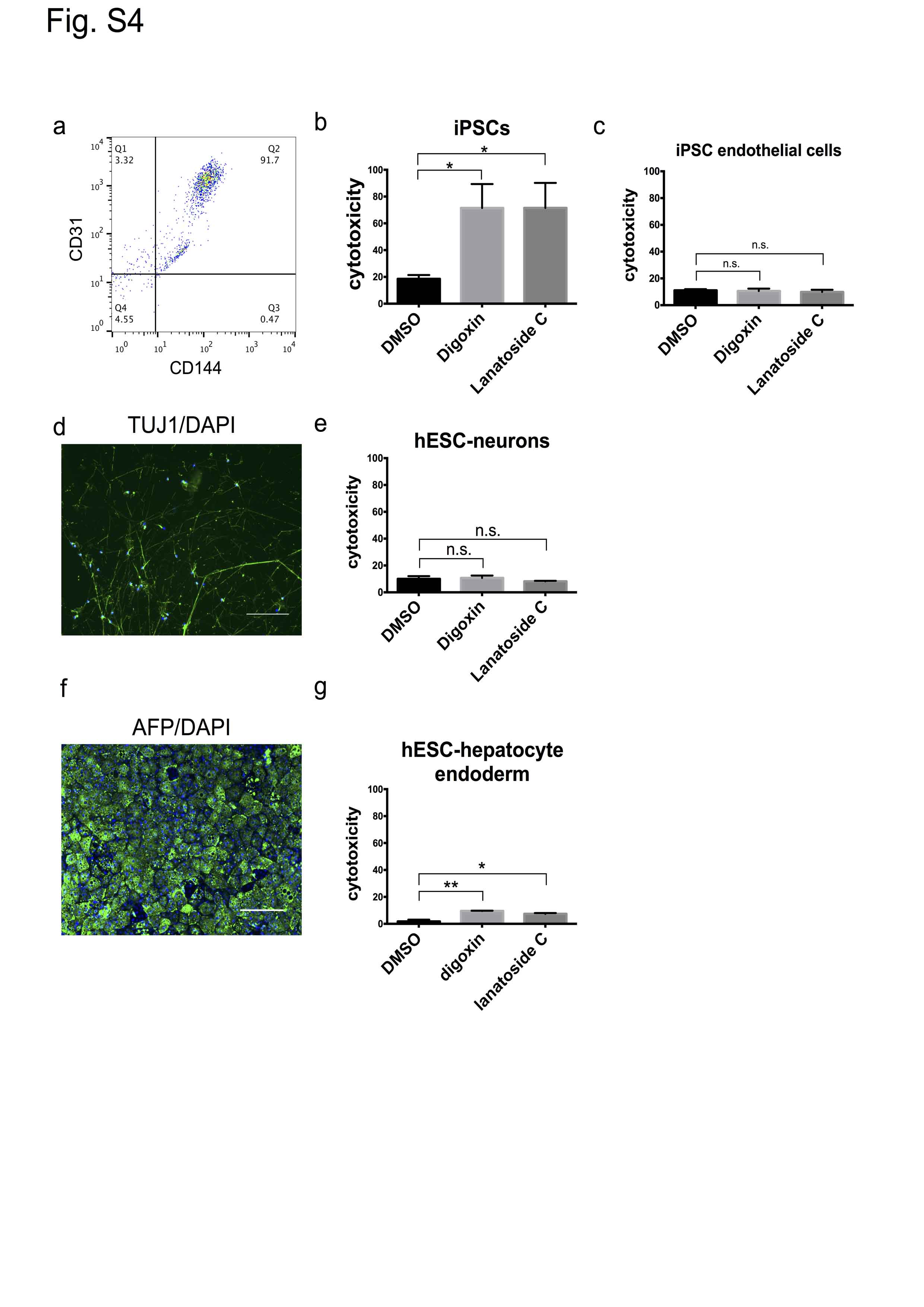
**

**Supplementary Figure 4. Cardiac glycosides did not or slightly affect the survival of hPSC derived cells.** (a) FASC analysis of the endothelial cell markers CD34 and CD144, which demonstrated over 90% CD34+/CD144+ population in hiPSC-endothelial cells. (b, c) cell were treated with 2.5 μM of digoxin and 2.5 μM lanatoside C for 24 hours, and the amount of released LDH was measured in the cytotoxic assay. Cardiac glycosides induced cytotoxicity in the undifferentiated hiPSCs but did not affect the survival of hiPSC-endothelial cells. (d) Immunofluorescence staining represented the neuron marker TUJ1 in hESC-neuron. Green: TUJ1; DAPI (blue): nucleus. (e) hESC-neurons were treated with 2.5 μM of digoxin and 2.5 μM lanatoside C for 24 hours, and cytotoxicity was not induced according to the measured released LDH. (f) Immunofluorescence staining represented the hepatocyte marker AFP in the hESC-hepatocyte endoderm. Green: alpha-fetoprotein; DAPI (blue): nucleus. (g) hESC-hepatocyte endoderm cells were treated with 2.5 μM digoxin and 2.5 μM lanatoside C for 24 hours, and cytotoxicity was slightly induced, as measured by the released LDH. Scale bar: 200 μm. **P<0.01; *P<0.05; n.s. not significant. Data are shown as the mean ± SD.

**
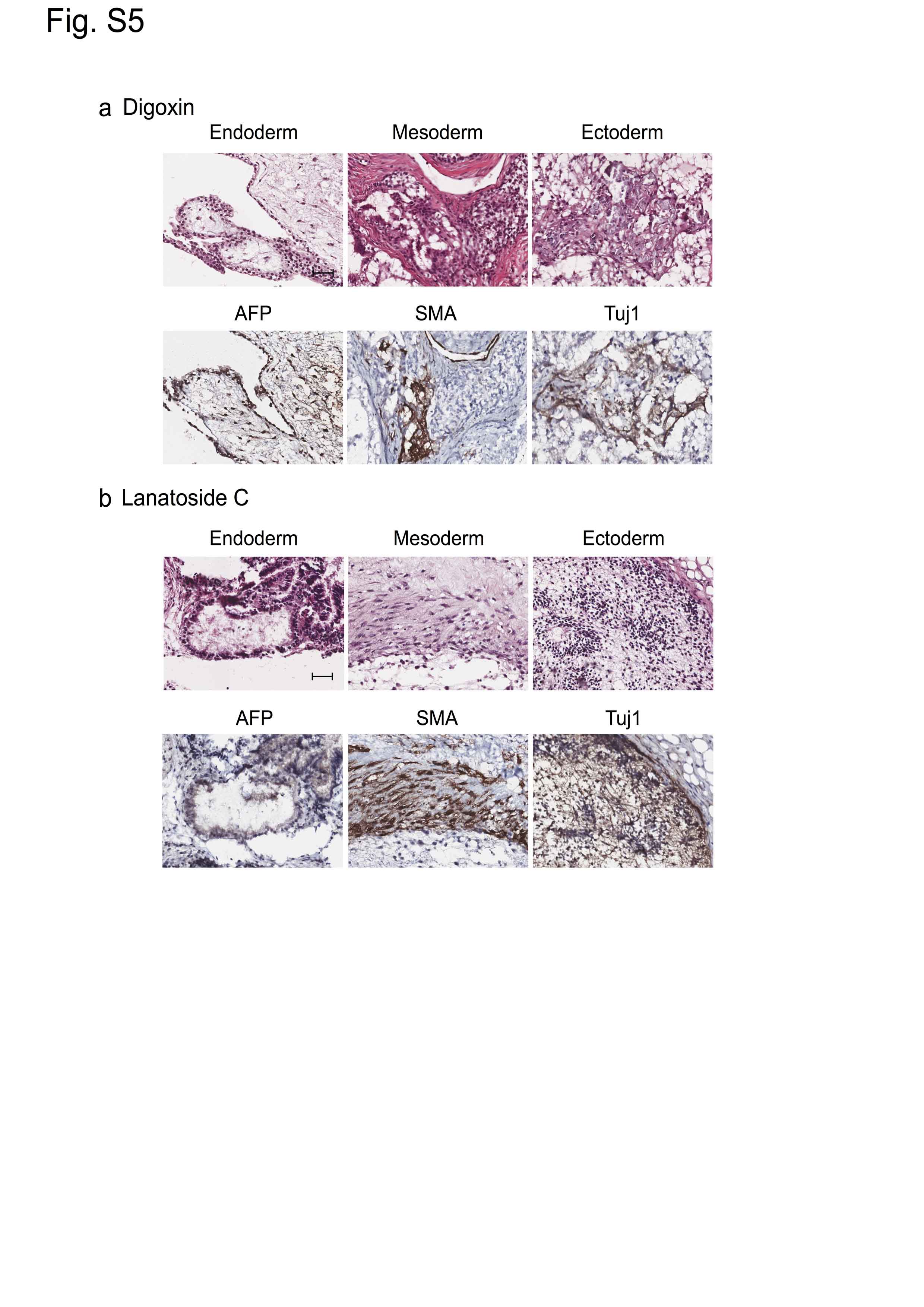
**

**Supplementary Figure 5. Immunohistochemistry images of cardiac glycoside-treated teratomas.** These images represent paraffin sections showing the three germ layer lineages of cardiac glycoside-treated teratomas. (a) Digoxin-treated teratoma sections were stained with H&E (top panel) and underwent IHC staining for the three lineage markers (bottom panel). (b) Lanatoside C-treated teratoma sections were stained with H&E (top panel) and underwent IHC staining for the three lineage markers (bottom panel). AFP (alpha-fetoprotein): endoderm marker. SMA (smooth muscle actin): mesoderm marker. Tuj1 (beta-III tubulin): ectoderm marker. Scale bar: 50 μm.

**
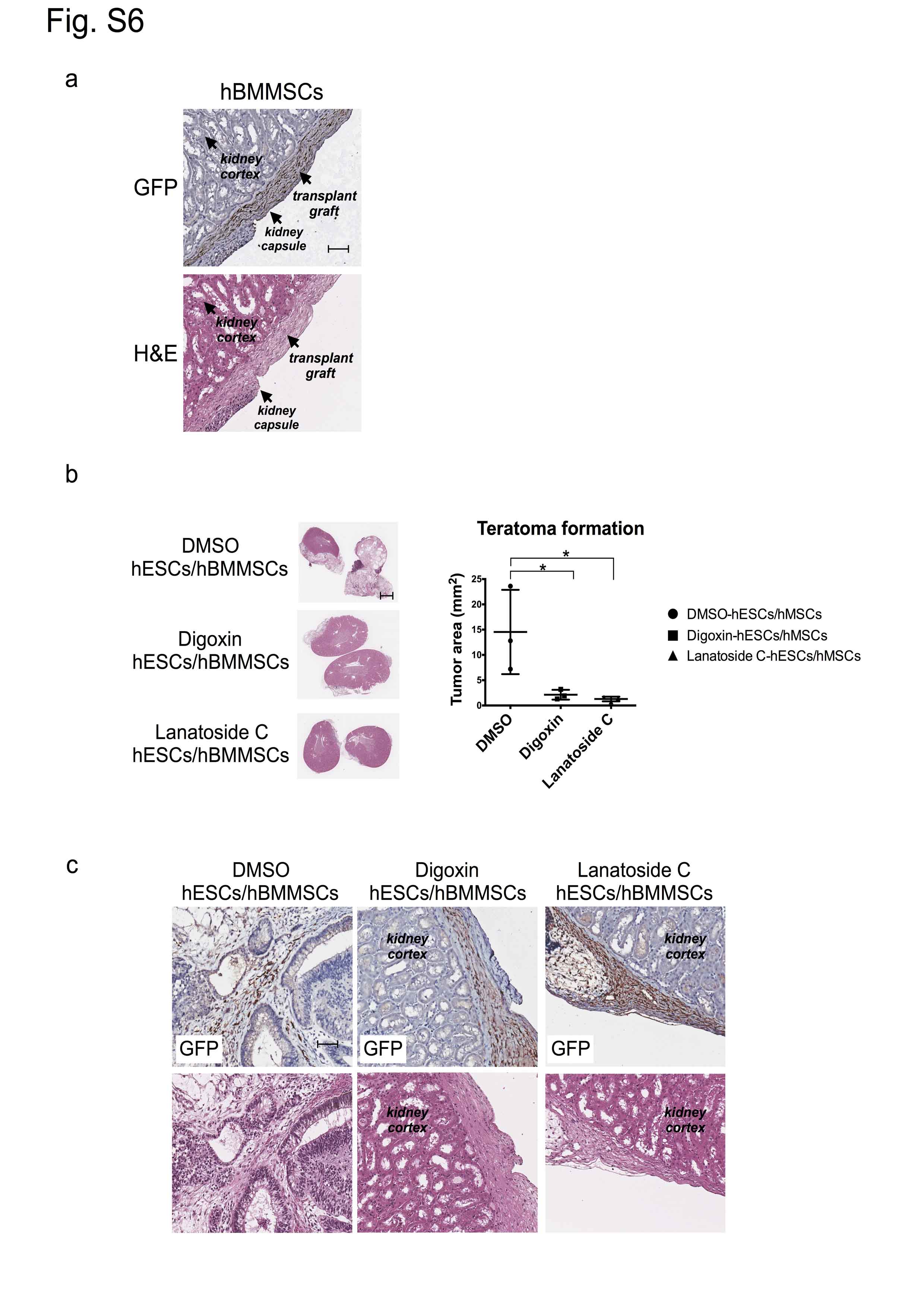
**

**Supplementary Figure 6. Drug-treated hBMMSCs remained in NSG mice after the cell transplantation.** (a) GFP-overexpressing hBMMSCs were injected under the kidney capsule, and hBMMSCs were stained with the GFP antibody to demonstrate that cells remain in the NSG mice. (b) Left panel: teratoma sections were obtained from mixtures of undifferentiated hESCs and GFP-overexpressing hBMMSCs that both were treated with DMSO, digoxin or lanatoside C under the kidney capsule in NSG mice. Right panel: tumor area of kidney tissue was quantified using image scope software. (n= 3) Scale bar: 2 mm. (c) Teratoma sections were stained with the GFP antibody (top panel) and H&E (bottom panel), which showed that the drug-treated hBMMSCs remained in the engraft site. Scale bar: 50 μm.


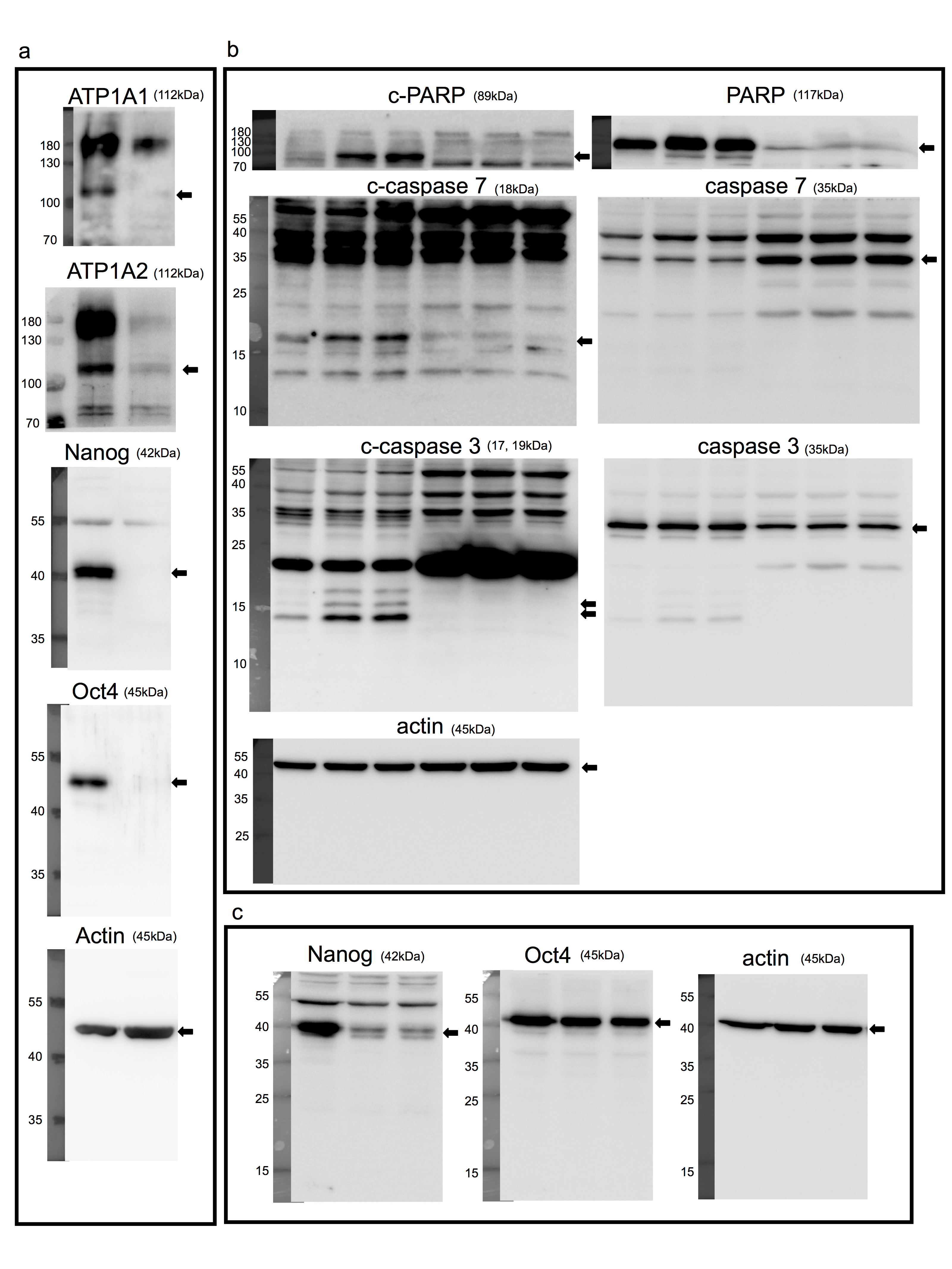


**Supplementary Figure 7. Original images of western blots were used in this manuscript.** (a) Represents the raw images in the Figure 1a. Samples were assayed in the 8 % SDS-PAGE. (b ,c) Represents the raw images in the Figure 2c and 2d. Protein samples in the (b) and (c) panel were assayed in the 12 % and 8 % SDS-PAGE respectively. We did not process brightness and contrast of all images and only cut into appropriate size for publishing.


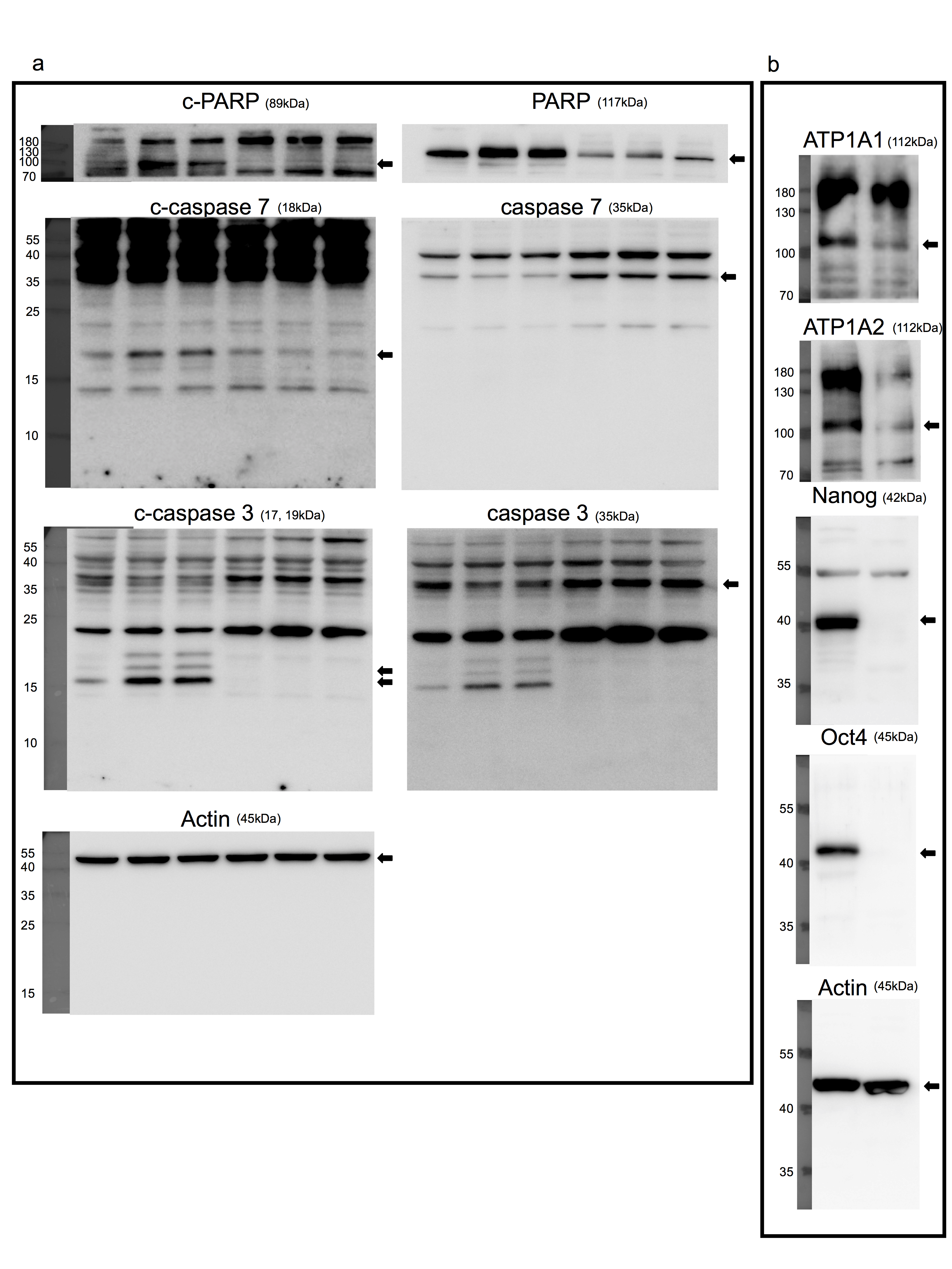


**Supplementary Figure 8. Original images of western blots were used in this manuscript.** (a,b) are the original images of Figure 4d and 4e. Protein samples in the (a) panel were assayed in the 12 % SDS-PAGE and and (b) panel were assayed in the 8 % SDS-PAGE. We did not process brightness and contrast of all images and only cut into appropriate size for publishing.
